# Naegeli-Franceschetti-Jadassohn syndrome: a systematic review of case studies

**DOI:** 10.3389/fmed.2025.1453172

**Published:** 2025-02-28

**Authors:** Hussain Haider Shah, Tooba Hussain, Arun Subash, Ramsha Abdul Qadir, Yashika Rajesh Meshram, Maryam Shahzad, Wania Sultan, Zeenat Hadi, Faiza Ashfaque, Zahra Anas, Sameer Abdul Rauf, Radeyah Waseem, Muhammad Sheheryar Hussain, Muhammad Abdul Wasay Zuberi

**Affiliations:** ^1^Department of Surgery, Dow University of Health Sciences, Karachi, Pakistan; ^2^Dr. Ruth K. M. Pfau, Civil Hospital Karachi, Karachi, Pakistan; ^3^Mahatma Gandhi Mission Medical College and Hospital, Aurangabad, India; ^4^Liaquat National Medical College, Karachi, Pakistan

**Keywords:** Naegeli-Franceschetti-Jadassohn syndrome, NFJS, dermatological disorders, clinical presentation, systematic review

## Abstract

**Introduction:**

Naegeli-Franceschetti-Jadassohn syndrome (NFJS), also known as Naegeli Syndrome, is a rare autosomal dominant ectodermal dysplasia characterized by mutations in the *KRT14* gene. These mutations disrupt ectodermal tissue development, leading to diverse clinical manifestations involving the skin, nails, teeth, and sweat glands.

**Methodology:**

A systematic search across PubMed, Google Scholar, European PMC, and Cochrane databases was conducted up to August 2023. Only case reports, case series, and original articles reporting cases were included.

**Results:**

This review incorporated 6 case reports, 2 case series, 3 original articles, and 1 editorials, encompassing 33 individuals diagnosed with NFJS. Key clinical features included extensive reticulate hyperpigmentation, palmoplantar keratoderma, and dental anomalies. Rarely reported findings, such as cerebellar fissures and generalized osteopenia, were noted in two cases. Treatment predominantly focused on symptomatic management using topical emollients and antioxidants.

**Conclusion:**

NFJS remains a diagnostic challenge due to its rarity and overlap with other pigmentary disorders. This review consolidates current knowledge to aid clinicians in recognizing and managing NFJS. Further research is needed to clarify its pathogenesis and explore targeted treatments.

**Systematic review registration:**

https://www.crd.york.ac.uk/prospero/display_record.php?RecordID=447267, identifier CRD42023447267.

## 1 Introduction

Naegeli-Franceschetti-Jadassohn syndrome (NFJS), also recognized as Naegeli Syndrome, is a distinctive autosomal dominant disorder that arises from mutations in Keratin 14 (KRT14) gene, disrupting the normal development and differentiation of ectodermal tissues. This rare genetic condition presents a range of symptoms primarily affecting the skin, nails, teeth, and sweat glands. Notably, a defining feature of NFJS is adermatoglyphia or loss of fingerprints. This absence is a cardinal diagnostic criterion, distinguishing NFJS from other conditions (1, 2).

The clinical features of NFJS present challenges and insights into genetic anomalies (3). Reticulate hyperpigmentation, marked by patterned pigmented patches on the skin, is a key characteristic. This pigmentation, appearing on the neck, chest, and abdomen during early childhood (3 months to 6 years), often fades after puberty and may resolve entirely by age 60 to 80. Localized pigmentation around the eyes and mouth is also possible (1, 4).

Individuals with NFJS often experience palmoplantar keratoderma, causing thickened skin on the palms and soles, reduced sweating, and difficulty with temperature regulation, significantly impacting their quality of life (5). Dental anomalies, such as enamel irregularities, vary in severity among patients. Additional skin symptoms, including blistering and nail dystrophy, further highlight the syndrome’s diverse clinical spectrum (1).

The genetic basis of NFJS lies in mutations affecting genes crucial for the development and function of ectodermal tissues. Among these, heterozygous nonsense or frameshift mutations in the E1/V1-encoding region of the KRT14 gene have been identified as key contributors, resulting in haploinsufficiency for KRT14, a structural protein essential for epithelial integrity. Previous studies have pinpointed the locus for NFJS on chromosome 17q, specifically between markers D17S933 and D17S1789/D17S934, with a maximum logarithm of odds score (LOD) score of 2.7 at D17S800, further corroborating the role of KRT14 mutations (1, 2, 4, 6–9). These genetic insights advance our understanding of the disorder’s complexities, offering the potential for enhanced diagnostic precision and the development of targeted therapeutic interventions in the future (1, 2, 4, 6–9).

This review systematically synthesizes data from published case reports and case series to provide an in-depth overview of NFJS. By summarizing its clinical manifestations, genetic basis, and management strategies, this study aims to enhance clinical understanding of NFJS and identify gaps in the current literature, highlighting areas for future research.

## 2 Methodology

This systematic review fully comply with the preferred reporting items for the systematic review and meta-analysis (PRISMA) 2020 statement (10). The protocol has been given the identification number CRD42023447267 and is recorded on the website PROSPERO.

### 2.1 Search and selection

Our electronic search encompassed databases such as PubMed, Google Scholar, European PMC, and Cochrane, spanning from their inception to August 2023. The search strategy employed MeSH terms and relevant keywords associated with “Naegeli-Franceschetti-Jadassohn syndrome.” Specifically, the search equation employed was “Naegeli-Franceschetti-Jadassohn syndrome OR NFJ Syndrome AND case reports AND case series.” To broaden our scope, we examined the references cited within the selected original articles, and we also conducted forward citation searches via the Web of Science to identify potentially relevant articles.

All articles were imported into Rayyan, and duplicates were systematically eliminated. Two authors (FA and ZH) independently evaluated the titles and abstracts of all retrieved articles, removing those that did not meet the predefined inclusion criteria. Full texts of the remaining articles were meticulously assessed against the established eligibility criteria. In cases of conflicts or discrepancies, discussions ensued, and a resolution was achieved through consultation with a third author (HS). The inclusion criteria encompassed case reports and case series written in English, targeting the general population affected by Naegeli-Franceschetti-Jadassohn syndrome. Study designs other than case reports and case series, as well as animal studies and articles in languages other than English, were excluded from consideration.

### 2.2 Data extraction

Two different researchers (FA. and ZH) carried out the process of data extraction on their own, and any discrepancies that arose were resolved by discussion with a third researcher (HS). The data collected from the eligible studies encompassed various aspects, including the primary author, publication year, journal title, study design, age and gender distribution of participants, inheritance pattern, the number of affected siblings or family members, laboratory findings, radiological observations, the onset of pigmentation changes, biopsy results, and the distribution of hyperpigmented and hypopigmented areas. The study’s primary outcomes were focused on genetic alterations, skin manifestations, hair abnormalities, nail characteristics, and dental anomalies. Additionally, secondary outcomes encompassed the fading of pigmentation, intellectual functioning, and lifestyle considerations.

### 2.3 Assessment of risk of bias

Using the JBI Checklist for Case Reports, two separate authors (FA and ZH) analyzed to determine the level of bias that existed among the studies that were included in the review. This checklist consists of eight distinct domains, each addressing specific aspects: (1) patient demographic characteristics, (2) patient history presented as a timeline, (3) current clinical condition on presentation, (4) description of diagnostic tests or assessment methods along with results, (5) clear depiction of interventions or treatment procedures, (6) description of post-intervention clinical condition, (7) identification and description of adverse events or (8) unanticipated occurrences, and provision of takeaway lessons. For each study, this tool was applied, and the presence of bias was categorized as yes, no, unclear, or not applicable, ultimately determining the level of bias risk as high, low, or with some concerns. Any inconsistencies that were discovered were discussed with a third author (HHS) to find a solution.

## 3 Results

### 3.1 Study selection

**FIGURE 1:**
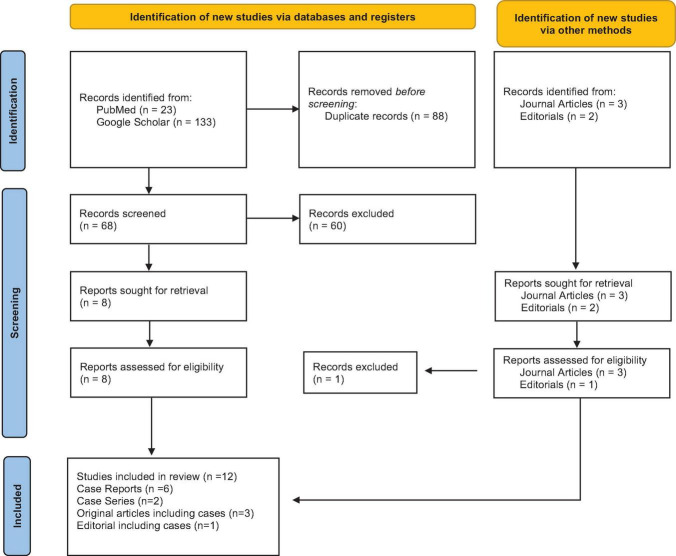
PRISMA flowchart outlining the study search.

In our systematic review, the search method yielded 156 records, of which only 68 were retrieved after deleting 88 duplicate articles. After screening the title and abstract, we eliminated 61 records, leaving us with 8 for the full-text screening. Furthermore, 5 items were retrieved, including 3 journal articles and 2 editorials reporting on cases, and were thus included. Finally, the systematic review contained 13 papers, of which 6 were recognized as case reports, 2 as case series, 3 as original pieces reporting cases, and 1 as editorials reporting cases (Figure 1).

### 3.2 Patient characteristics

The features of the studies that were considered in this review can be seen in Table 1. The research consisted of a total of 33 individuals who had been diagnosed with NFJ syndrome. The review contained a total of 6 case reports, 2 case series, 3 original articles reporting cases, and 1 editorial reporting cases. A total of 33 patients were reviewed, including 18 females (54.5%) and 15 males (45.5%). The age of diagnosis ranged from 2 to 66 years. Most cases 90.0% (*n* = 30) exhibited autosomal dominant inheritance, while 3% (*n* = 1) showed autosomal recessive inheritance, and 6.9% (*n* = 2) had unspecified inheritance patterns. The age of NFJ syndrome diagnosis exhibited a broad range, spanning from the early onset at 2 years of age to a relatively late presentation at 66 years of age.

**TABLE 1:** Study Characteristics of the included studies in our systematic review.

References	Study design	Age	Gender	Pattern of inheritance	Number of siblings/ relatives affected	Hyper pigmen tation/ hypopi gmen tation areas	Biopsy finding	Genetic changes	Hair nails, teeth and skin changes	Lab findings	Radiolo gical findings	Unique feature	Onset of pigmen tation	Treatment given
Sanodia et al. (8)	Case report	16	Female	Autosomal dominant pattern	Not mentioned	Reticulate hyperpigmen tation is generalized and involves the whole body. It is more prominent over the neck, chest, hands, and legs. Tongue’s dorsal aspect shows diffuse pigmentation.	Specimen taken from the left side of the neck showed basket wave pattern hyperkeratosis with diffuse pigmentation of the basal layer. The upper dermis showed pigment incontinence.	Dominant mutations in the nonhelical E1/V1 domains of keratin 14 (KRT14) gene.	Hair shows mild pigmentary dilution with patchy brown discoloration. Nail changes include nail dystrophy. Reticulate hyperpigmen tation and xerosis of the skin are observed, dental changes including yellowish discoloration, abnormal dentition, and enamel defects.	Complete hemogram, random blood sugar, liver function test, renal function test, and urine analysis were within normal limits. The article does not provide more detailed lab findings.	Not mentioned	The involvement of proximal and distal interphalan geal joints, which is a unique feature,	Reticulate hyperpigmen tation starts around the age of 2 years without a preceding inflammatory stage. The pigmentation increases gradually during the first 10 years of life. Fading of pigmentation starts around the age of 15 years.	No specific treatment exists for NFJS, except for symptomatic management of some of the associated conditions. Genetic counseling should be offered to affected individuals planning future children
Tubaigy et al. (27)	Case report	23	Male	Autosomal dominant pattern	2 brothers aged 35 and 40 also displayed similar features of NFJ syndrome.	The hyperpigmenta tion in NFJ syndrome occurs primarily on the abdomen and around the eyes and mouth, with the neck, trunk, axillae, groins, and proximal extremities and flexures more variably involved. Some patients develop bullae on the feet during the newborn period.	Not mentioned	Not mentioned	Skin changes with hypo- and hyperpigmen tation, hypo-hidrosis, dystrophy of the nails, and diffuse thickening of the palms and feet.	Not mentioned	Not mentioned	Absence of fingerprints (dermatogly phics), reticulate hyperpigmen tation,	The pigmentation starts around the age of 2 years without a preceding inflammatory stage and may fade after puberty, often disappearing by around the age of 60.	Not mentioned
Shah et al. (14)	Case report	27	Male	Autosomal dominant	Not mentioned	Reticulate hyperpigmen tation involving the whole body, especially dense over the palms and soles, and a reticulate pattern on elbows, knees, dorsal aspect of the tongue, neck, trunk, proximal extremities, axillae, and groin.	Biopsy specimen from a hyperpigmen ted macule on the back showed findings of epidermal atrophy, vacuolar degeneration, hyperpigmen tation of the basal layer, and pigmentary incontinence. The dermis showed perivascular chronic inflammation, increased dermal fibrosis, with the presence of dermal melanophages.	Mutations in the KRT14 gene are associated with Naegeli-Franceschetti-Jadassohn syndrome (NFJS).	The patient had mild pigmentary dilution of hair, severe nail dystrophy, reticulate hyperpigmen tation, and atrophy of the skin over the dorsal aspect of hands and feet.	Baseline investigations were within normal limits except for a slightly lower hemoglobin level of 9 g/dL. Blood arsenic level was below detection limit. Molecular analysis for KRT 14 gene mutation using genomic DNA from the blood of the patient was done by the method of PCR-bidirectional sequencing of exons 1, 4, and 6. It showed absence of mutation in the three exons (1, 4, and 6), which ruled out epidermolysis bullosa simplex (which is also caused by the mutation in this gene); however, possibility of NFJS/DPR cannot be ruled out on the basis of this study.	Not mentioned	Pigmentary dilution of hair.	Reticulated hyperpigmen tation usually develops by the age of 2 years, without a preceding inflammatory stage. Hyperpigmen tation often fades after puberty and may eventually disappear.	The patient was advised to have strict photo protection, teeth care, avoiding strenuous activity, maintaining adequate hydration, given oral antioxidants, and topical emollients. Doxycycline has been found to interfere with tumor necrosis factor-alfa–mediated signaling and apoptosis *in vitro* and may have a role in future treatment.
Itin et al. (3)	Case reports	The pedigree includes 62 members with 14 affected patients. CASE 1 age 12 years, CASE 2 age 9 years, CASE 3 age	6 males, 8 females	Autosomal dominant	The pedigree includes 62 members with 14 affected patients.	The reticulate pigmentation starts at about the age of 2 years and fades after puberty, eventually disappearing completely in old age.	Not mentioned	Not mentioned	Hair, nails, and skin changes: The patients lack dermatogly phics, have diffuse palmoplantar keratoderma that may coexist with punctate keratoses, and experience hypohidrosis. Teeth are always severely affected, leading to early total loss	Not mentioned	Not mentioned	Congenital malalignment of the great toenails was found in four patients, which was not previously described in association with the NFl syndrome	The reticulate pigmentation starts at about the age of 2 years	Not mentioned
Sparrow et al. (16)	Case series	Case 1 = 7 years, Case 2 = 37 years, Case 3 = 48 years, Case 4 = 17 years, Case 5 = 18 years, Case 6 = 21 years, Case 7 = 47 years	Case 1 female, Case 2 male, Case 3 female, Case 4 female, Case 5 female, Case 6 male, Case 7 male	Autosomal dominant male to male no consanguinity	Seven individuals from one family are recorded to have the syndrome	The hyperpigmenta tion is most marked around the neck, on the upper trunk, and in the flexures. It is also present on the sides of the neck, in the axillae, and on the trunk.	The biopsy findings include pigment-containing melanophores in the upper dermis, variable atrophy and edema, dyskeratotic cells, and increased melanin in the epidermis	Not mentioned	The syndrome is characterized by hyperpigmen tation, hypohidrosis, dystrophy of the nails, punctate keratoses of the palms and soles, hypoplasia of the dermatogly phics, atrophic changes of the skin of the face, bullae on the feet, and dental anomalies. The nails show onycholysis, subungual hyperkeratosis, and slight thickening.	The study includes tests of sweating, which show a variable but slight decrease in the numbers of sweat glands and a more severe defect of function.	Not mentioned	Anhidrotic ectodermal dysplasia, epidermal ridge atrophy	The hyperpigmen tation begins in early childhood and fades during adolescence.	Not mentioned
									The palms and weight-bearing areas of the soles show multiple small punctate keratoses. The dermatogly phic pattern is hypoplastic on the finger pulps and to a lesser extent on the palms and soles					
Tzermias et al. (13)	Case report	19	Female	Autosomal dominant	The patient’s father and grandfather showed generalized reticular pigmentation, which had faded in adulthood, as well as the same type of palmoplantar hyperkeratosis; both died during the patient’s childhood, the former aged 37 from acute ischemic heart disease, and the latter aged 64 from an acute hepatic disease	Faint generalized reticular hyperpigmen tation, more intense and accompanied by marked milia formation in the flexural areas (neck, axillae, cruro-inguinal, antecubital and popliteal) as well as on the waist and lateral abdominal regions. Rare hypopigmented macules of various sizes were scattered among the hyperpig mented lesions	Histopatholo gical findings were not distinctive, as there was only slight hyperpigmen tation of the basal layer of the epidermis in areas with faint reticular hyperpigmen tation, such as the calf, in addition to occasional dermal melanophages in an area with intense hyperpigmen tation, such as the cruro inguinal region where milia formation was also observed. Marked compact orthokeratosis was seen in the palmar area.	Genetic investigation revealed a normal 46, XX chromosomal composition with no chromosomal abnormalities or markers	Diffuse mild palmoplantar hyperkeratosis, dental abnormalities (yellowish coloration and an enamel defect necessitating dental porcelain prostheses on five of her front teeth), slight yellowish hue to the nails and partial flattening of dermatog lyphics were also observed	Normal routine hematological and biochemical tests, hormone tests, chest X-ray, ECG, and EEG. The partial hypoplasia of dermatog lyphics was identified using the skin surface replica method. No functional disorder of perspiration was identified using the acetylcholine iontophoresis test evaluated with the starch iodine method in combination, with the plastic impression technique	Normal chest X-ray	Generalized dark brown reticular hyperpigmen tation and diffuse mild palmoplantar hyperkeratosis and milia formation	From birth	Not mentioned
Kudo et al. (15)	Case report	4	Male	Not mentioned	His father had a similar pigmentation when he was a child. The boy’s heterozygous twin-sister did not	brown, reticulate, pigmented macules on the body, except for the face and the palmoplantar surface. On the anterior aspect of the neck, from the arm pits to the upper arms and from the lower abdomen to the thighs, brown-colored, hyperpigmented macules were intermingled with fine flesh-colored spots. Brown black macules were present on the gingiva and buccal membrane. Periungual areas of fingers and toes were also pigmented	Light microscopic examination of biopsy specimens from the thigh revealed slight hyper melanosis of the basal layer, an increased number of melanophages in the papillary dermis, and some eosinophilic degenerating cells in the epidermis	Chromosome analysis showed a normal karyotype	pigmented, dry skin, covering nearly the whole body, without any preceding inflammatory changes. No deformity of the nails or teethwas found.	Complete blood cell counts and routine laboratory chemistry values were within normal limits. Among endocrino logic tests, thyroid-stimulating hormone (TSH) showed a high value of 17.1 pU/mL (normal 0.4–3.3), although T3 and T4 values were within the normal range.	The bone age of the hand was 3 years.	Not mentioned	2 or 3 years	For subclinical hypothyroidism thyroxine was given
										The release of TSH, adrenocortico tropic hormone (ACTH), growth hormone (GH), prolactin, luteinizing hormone (LH), or follicle stimulating hormone (FSH) after appropriate stimuli was not impaired. The sweat test, using the method of Sato, revealed a positive reaction only in the axilla, finger and toe tips, buttocks, and part of the back.				
Belligni et al. (11)	Original article	Case 1 8 years Case 2 3 years	Both males	Not mentioned	Case 1 elder sister had bilateral hearing lossCase 2 Not mentioned	Case 1 A reticulate hyperpigmen tation was evident over his whole body, and most marked on forehead, nose, thorax and abdomen, and which was raised on the limbs and hypopigmented spots on face and limbs Case 2 A mottled skin hyperpigmen tation with small areas of depigmentation was spread all over the body, less pronounced on the cheeks, the nose and around the eyes	Case 1 A skin biopsy showed a completely normal dermis and subcutis, and no increased sensitivity for UV light was found. Case 2 A skin biopsy detected apoptosis in the basal layer and mild degree of pigment incontinence	Case 1 Standard chromosome analysis showed a normal male karyotype (46, XY) and CGH array (BlueGnome CytoChip; 0.7 Mb) failed to show an imbalance. KRT14, TERC and TINF2 (exon 6) analysis (for Autosomal Dominant dyskeratosis congenita) did not detect any mutations. Case 2 Classical chromosome analysis showed a normal male karyotype (46, XY) and CGH array (BlueGnome CytoChip; 0.7 Mb) failed to show an imbalance.	Case 1 His skin was otherwise dry, and careful inspection of the tips of his fingers showed markedly under developed fingerprints. Hair and nails were normal. Hands and feet were normally shaped. He had poor anterior teeth thought to represent bottle cariescase 2 His skin was dry, and sweating was decreased.	Case 1 immunologic studies, which showed transient lowered levels of IgG and IgA and neutropenia. Bone marrow analysis confirmed the neutropenia without any other abnormalities. Plasma and urine amino acids profile, urine organic acids, free and acylcarnitine and plasma very long chain fatty acids profile, blood lactate/pyruvate ratio, urine NAG/creatinine ratio, purine metabolisms, thyroid hormone analysis all gavenormal results	Case 1 Brain MRI showed prominence of cerebellar fissures and fourth ventricle, otherwise the brain had a normal anatomy. A skeletal survey and abdominal ultrasound gave normal results.case 2 A skeletal survey showed generalized osteopenia, a delayed bone age, and some tapering of distal phalanges at hands and feet	Case 1 He showed a high nasal bridge, prominent nose, and his columella extended well below the alae. His ears showed additional soft skin covering the upper parts, as can be seen after bleedings in ear cartilage, but significant ear trauma were denied by the parents. Bilateral pale optic disks case 2 He had a narrow face, upslanted palpebral fissures, proptosis, epicanthic folds,	Case 1 18 months Case 2 first months	Not mentioned
								Microsatellite analysis excluded uniparental disomy of chromosome 7 and 15 and methylation sensitive PCR of ICR1 and ICR2 gave normal results. Sister chromatid exchanges analysis did not show any significant raised levels of spontaneous exchanges and there was nonevidence of radiosensiti vity in chromosome breakage studies in fibroblasts. KRT14 analysis did not detect any mutations.	Fingerprints were essentially absent; nails were slowly growing, small and thin, especially on the halluces. Hair was sparse, thin and silvery blond He had marked caries, but enamel was thought to be normal. He missed 4 elements. The shapeof teeth was normal.	Case 2 Leukocyte counts, plasma levels of cholesterol, triglycerides, alkaline phosphatase, electrolytes, thyroid hormone, lactate, carnitine, amino acids, all with normal results. No abnormal glycoforms were found by transferrin electro phoresis		broad nasal ridge and tip, long philtrum with a thin upper vermilion and a small chin		
Hussain SM et al. (2)	Case report	18-year	Male	Autosomal dominant	Not mentioned	Brownish black reticular marking over the neck	Not mentioned	KRT14 gene located on chromosome 17q12-21; frame shift or nonsense mutation in KRT14 gene leading to early termination of translation or mRNA degradation	Marfanoid features, smooth skin of palms, abnormal dentition	Not mentioned	Not mentioned	Lack of dermatoglyphics (fingerprints)	Not mentioned	Not mentioned
Itin et al. (12)	Letter which includes case report	66 year	Female	Autosomal dominant	Not mentioned	at the age of 65 complete resolution of reticular pigmentation	Not mentioned	Keratin 14 mutation confirmed previously	impressive enamel defects and total prostheses necessary at age of 30, patient had palmoplantar keratoderma, but nails are unaffected	pilocarpine test was weakly positive	Not mentioned	Not mentioned	at the age of 65 complete resolution of b pigmentation occur	Not mentioned
Ralser et al. (9)	Journal article which included case reports.	Case 1 = 21 years Case 2 = 18 years	Female	Autosomal dominant	Index case 1 21 years old and her sister case 2 18 years old, also 9 other members of family were affected including their father and grandfather	hyperpig mented lenticular-sized spots in the axillary and inguinal regions, and mild palmoplantar keratoderma	Not mentioned	Sequencing of KRT14 revealed a c.54C > A substitution, leading to a premature stop codon at position 18 [p.(Cys18)]. The mutated sequence is shown in comparison with the wildtype (WT) sequence. Illustration of haplotypes on chromosome 17 in the investigated affected individuals from both families	Skin erosions and blistering in Case 1 postpartum. hyperpig mented lenticular-sized spots in the axillary and inguinal regions, and mild palmoplantar keratoderma. The index patient had developed blistering and peeling skin a few hours postpartum, which had healed within the first weeks of life without scarring or recurrence. presented with anhidrosis.	Diagnostic DNA analysis was performed. haplotype analyses were performed in the index patient and her sister,	Not mentioned	Not mentioned	Not mentioned	Not mentioned
Lugassy et al. (1)	Journal article which included a case report	8	Female	Autosomal dominant	Not mentioned	Mottled hyperpigmen tation most prominently over the trunk	Skin biopsy showed several foci of apoptosis in the basal cell layer, numerous melanophages in the upper dermis	Heterozygous single-nucleotide deletion at position 29 of the complemen tary DNA (cDNA) nucleotide sequence	Skin atrophy over the knuckles and palms, slowly growing hair, abnormal dentition Mottled hyperpigmen tation of the skin, predomi nantly on the trunk, epidermal ridge dissociation of finger and toe tips, skin atrophy over the knuckles and palms, hypohidrosis, abnormal dentition, slowly growing hair, occasional skin blistering on the arms and legs	Sequenced the entire coding region of the KRT14 gene (including exon–intron boundaries) and identified a previously unknown, heterozygous single-nucleotide deletion at position 29 of the complemen tary DNA (cDNA) nucleotide sequence (c.29delC, NM_000526)	Not mentioned	Not mentioned	Not mentioned	Not mentioned

Onset of Pigmentation was primarily noted at around 2 years of age, although cases were documented with earlier onset as early as 18 months (11) and later onset as late as 65 years of age (12) in two individuals.

### 3.3 Clinical presentation

The clinical presentation of the NFJ syndrome prominently featured extensive reticulate hyperpigmentation affecting various regions of the body. 90.9% (*n* = 30) cases displayed pronounced hyperpigmentation notably around the neck, upper trunk, chest, flexural areas such as axillae, cruro-inguinal, antecubital, and popliteal regions, as well as the proximal extremities, and the groin.

**FIGURE 2:**
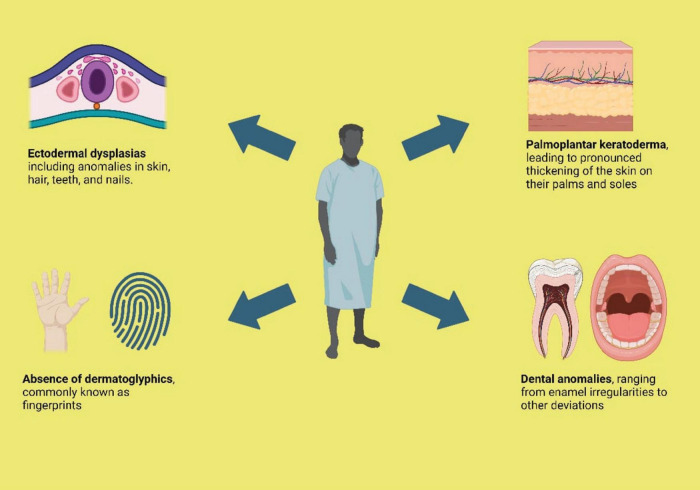
Common clinical features of NFJ syndrome.

Additionally, reticulate hyperpigmentation was sporadically observed in the waist, lateral abdominal area in 12.1% patients (*n* = 4), and the dorsal aspect of the tongue (8) in 3% cases (*n* = 1). Moreover, the palmar and plantar surfaces, including periungual areas of fingers and toes, also exhibited this hyperpigmentation along with palmoplantar keratoderma. In one particular case (*n* = 1), the patient also manifested reticulate hyperpigmentation on the forehead, nose, and cheeks (11). Furthermore, a rare occurrence of hypopigmented macules was noted in one case (*n* = 1), primarily around the hyperpigmented lesions in the abdominal and flexural regions in one particular case (13). Figure 2 Illustrates the common clinical features of NFJ syndrome.

Teeth, nail and hair changes were also observed in the patients. Dystrophy, onycholysis, subungual hyperkeratosis with slight yellowish hue of the nails was a prevalent clinical finding that affected patients (*n* = 27, 81.8%). Abnormal dentition including yellowish discoloration, abnormal dentition, and enamel defects were seen in patients (*n* = 25, 75.8%). Two cases reported poor anterior teeth representing bottle caries as well as impressive enamel defects requiring total prostheses at a young age (11, 12). Hair changes included mild pigmentary dilution with patchy brown discoloration (1, 3, 8, 11, 14). One of the cases in a case series reported sparse, thin hair which had turned silvery blond (11).

Concurrently with reticulate hyperpigmentation, 81.8% participants displayed palmoplantar keratosis (*n* = 27), 72% coupled with hypo hydrosis (*n* = 24) . 32.4% of participants exhibited a hypoplastic dermatoglyphic pattern and adermatoglyphia (*n* = 11). Additionally, a small group of participants also displayed Pigmentary dilution of hair with brown patches (*n* = 3). Figure 2 shows common clinical features of NFJ syndrome.

### 3.4 Management

Currently, no established treatment protocols are available with regards to the NFJ syndrome. Management primarily focuses on addressing associated conditions. Most case reports and series included in this systematic review did not provide specific treatment details. However, a single case report described the management of NFJ Syndrome in which the patient was presented with extensive reticulate hyperpigmentation. The patient was counseled to avoid vigorous physical activity, maintain proper hydration, and was administered oral antioxidants and topical emollients to support skin health (14). In one case, laboratory results revealed elevated TSH levels, leading to the prescription of thyroxine for subclinical hypothyroidism (15).

### 3.5 Radiological findings

The majority of case series and case reports analyzed in this systematic review did not incorporate radiological findings. Only one case series contained radiological findings. Among the two cases within case series (11), the first case exhibited a brain MRI displaying prominence of cerebellar fissures and the fourth ventricle, with an otherwise normal brain anatomy. A skeletal survey and abdominal ultrasound yielded normal results. In the second case (11), a skeletal survey revealed generalized osteopenia, delayed bone age, and tapering of distal phalanges in the hands and feet.

### 3.6 Histopathology

Most of the case reports and case series mentioned histopathology. (*n* = 15) of patients had their biopsy specimens taken from the skin in the neck, trunk, thigh, calf, and palmar surfaces or directly from the hyperpigmented macule. In most of the case reports diffuse pigmentation and pigment incontinence in the basal layer were common (*n* = 12, 36.4% cases). Sanodia et al also reported a basket weave pattern of basal layer with diffuse pigmentation (8). Other case reports 18.2% (*n* = 6) demonstrated epidermal atrophy, vacuolar degeneration, edema, dyskeratotic cells, eosinophilic degenerating cells, and basal cell apoptosis (1, 8, 11, 14–16). Additionally, melanin-containing melanophores in the upper dermis were visible as well as melanophages in the papillary dermis of intensely hyperpigmented areas (1, 13–16). One case report also observed orthokeratosis in the palmar area (13). Only one case (3%) in a case series of two cases showed no abnormal histopathology (11).

The findings and percentages of cases reported are shown in Table 2.

**TABLE 2:** Key findings and percentages in NFJ syndrome cases reported.

Category	Details	Percentage (*n*)
Gender distribution	Females	54.5% (18)
	Males	45.5% (15)
Inheritance patterns	Autosomal Dominant	90.% (30)
	Autosomal recessive	3.0% (1)
	Unspecified	6.9% (2)
Clinical features	Reticulate hyperpigmentation	90.9% (30)
	Lateral abdomen or tongue hyperpigmentation	11.8% (4), 2.9% (1)
	Nail dystrophy	79.4% (26)
	Abnormal dentition	73.5% (24)
	Palmoplantar keratosis	79.4% (26)
	Hypohidrosis	70.6% (23)
	Hypoplastic dermatoglyphics	32.4% (10)
	Pigmentary hair dilution	9.1% (3)
Histopathology	Diffuse pigmentation in basal layer	36.4% (12)
	Melanophages in papillary dermis	18.2% (6)
	Vacuolar degeneration, dyskeratotic cells	18.2% (6)
	Orthokeratosis in palmar area	3.0% (1)

### 3.7 Genetic changes

The majority of case reports included in our review discuss the genetic changes associated with NFJS syndrome. Among these cases, the most common mutation identified in NFJS syndrome affects the KRT14 gene, primarily in the non-helical E1/V1 domains of keratin 14 (2, 8, 12, 14). In one instance, a unique mutation was uncovered through PCR amplification and direct sequencing, revealing a homozygous one-base pair deletion in exon 4, labeled as c.827delC, along with a reduction in KRT14 mRNA levels (2). In separate investigations involving two case reports and one case series, genetic analysis indicated a normal 46, XX chromosomal composition without any chromosomal abnormalities or markers (11, 13, 15). Another noteworthy mutation in the KRT14 gene involved a c.54C > A substitution, resulting in a premature stop codon at position 18, as reported by Ralser et al. (9). Additionally, Lugassy et al. demonstrated a heterozygous single-nucleotide deletion at position 29 of the complementary DNA (cDNA) nucleotide sequence (1).

### 3.8 Quality assessment

To carry out a comprehensive analysis of the studies’ level of quality, the critical appraisal checklist for case reports developed by the Joanna Briggs Institute was utilized (17). This systematic review includes several studies, a major portion of which lacked information regarding therapy, management, post-interventional results, and post-interventional adverse events. The quality evaluation table for the studies that we included may be seen in Table 3.

**TABLE 3:** Quality Assessment table of included studies according to JBI checklist.

References	Were the patient’s demographic characteristics clearly described?	Was the patient’s history clearly described and presented as a timeline?	Was the current clinical condition of the patient on presentation clearly described?	Were diagnostic tests or assessment methods and the results clearly described?	Was the intervention(s) or treatment procedure(s) clearly described?	Was the post-intervention clinical condition clearly described?	Were adverse events (harms) or unanticipated events identified and described?	Does the Case Report provide takeaway lessons?
Sanodia et al. (8)	No	Yes	Yes	Yes	No	No	No	Yes
Tubaigy et al. (27)	Yes	Yes	Yes	No	No	No	No	Unclear
Shah et al. (14)	Yes	Yes	Yes	Yes	Yes	No	No	Yes
Itin et al. (3)	Yes	Yes	Yes	No	No	No	No	Yes
Sparrow et al. (16)	Yes	Yes	Yes	Yes	No	No	No	Unclear
Tzermias et al. (13)	Yes	Yes	Yes	Yes	No	No	No	Yes
Kudo et al. (15)	Yes	Yes	Yes	Yes	No	No	No	Yes
Belligni et al. (11)	Yes	Yes	Yes	Yes	No	No	No	Yes
Hussain SM et al. (2)	Yes	No	No	No	No	No	No	Unclear
Itin et al. (12)	Yes	Yes	Yes	Yes	Unclear	No	No	Yes
Ralser et al. (9)	Yes	Yes	Yes	Yes	No	No	No	Yes
Lugassy et al. (1)	Yes	Yes	Yes	Yes	No	No	No	Yes

## 4 Discussion

Our review aims to analyze and highlight the clinical manifestations and genetic basis of NFJ syndrome, offering insights to clinicians and researchers. As far as we know, this is the first systematic review of NFJ syndrome. This work consolidates existing knowledge while addressing gaps in understanding this rare condition. NFJ syndrome is a rare autosomal dominant form of ectodermal dysplasia affecting various body parts such as the skin, sweat glands, nails, hair, and teeth. The estimated incidence is one case in two to four million population. The syndrome is allelic to dermopatia pigmentosa reticularis (DPR) (18) (MIM125595).

The first description of the syndrome dates back to 1927 when Naegeli described a Swiss family consisting of a father and two daughters. Later on, in 1954, Franceschetti and Jadassohn reexamined the family, while Itin et al. (3) expanded the pedigree to include six generations with 14 affected individuals. Similarly, Sparrow et al. conducted a case series of seven patients with suspected NFJS syndrome and found that the findings were closely related to dyskeratosis congenita (DKC) which is commonly inherited as an X-linked recessive disorder, thus making it different from NFJS (14, 16).

NFJ syndrome shares overlapping features with other reticulate pigmentary disorders, such as DPR and DKC. However, NFJ is distinct in its fading pigmentation, absence of leukoplakia, and associated dental anomalies (19). According to Lugassy et al., both NFJ syndrome and DPR are allelic diseases resulting from dominant mutations in the KRT14 gene, located on chromosome 17q11.2–q21, which encodes keratin 14 (5). This gene is essential for the production of keratin 14, a protein that plays a critical role in the structure and function of the skin, hair, and sweat glands. Studies have identified heterozygous nonsense or frameshift mutations in KRT14 that segregate with the disease in affected families. These mutations are located in the non-helical head (E1/V1) domain of the keratin 14 protein, as opposed to mutations in the central α-helical rod domain, which are associated with epidermolysis bullosa simplex. NFJ syndrome and DPR are linked to mutations in the non-helical E1/V1 domains of KRT14 (5, 18). While both disorders share a common genetic origin, they present with distinct clinical manifestations. NFJ syndrome is characterized by epidermal hyperkeratosis and pigmentation changes, while DPR shows greater emphasis on diffuse keratoderma and palmoplantar abnormalities, with less involvement of the epidermal features seen in NFJ syndrome (5, 18). Furthermore, a deficiency in keratin 14 increases the likelihood of epidermal cells undergoing apoptosis. This loss of cells disrupts the normal development and structure of ectodermal tissues, leading to the skin and nail abnormalities that are hallmark features of NFJS/DPR (20, 21).

Our findings confirm that NFJ syndrome is linked to mutations in the KRT14 gene, particularly within the E1/V1-encoding region, generating a premature termination codon (PTC) close to the KRT14 translation initiation site (5). Those mutations result in a Keratin 14 haploinsufficiency, leading to increased epidermal cell susceptibility to tumor necrosis factor-alpha (TNF-a) mediated apoptosis resulting in developmental anomalies such as enamel defects and early caries in teeth. The same mechanisms likely contribute to potential bone involvement, although further research is needed to elucidate this aspect fully (22). The pigmentation has a brown or gray-brown reticulate pattern, starting at about 2 years of age without a preceding inflammatory stage. It may fade after puberty and often disappears by about the age of 60. Pigmentation occurs primarily on the abdomen and around the eyes and mouth, with the neck, trunk, axillae, groins, and proximal extremities and flexures more variably involved. Some patients develop bullae on the feet during the newborn period (3).

Affected individuals may also display palmoplantar hyperkeratosis, brittle fingernails, and in some cases misalignment of the great toenails (23). Among the most distinctive characteristics of this syndrome is the complete absence of the patterned ridges on the skin of the hands and feet, called dermatoglyphics that are the basis for each person’s unique fingerprints (24, 25).

A significant difference between DPR and NFJS is that reticulate pigmentation lasts until adulthood, and there is alopecia and no dental abnormalities. Although these conditions have similar skin changes to DKC and Poikiloderma Clericuzio, differential diagnosis can be made based on factors such as leukoplakia and bone marrow dysfunction in DKC and features such as telangiectasias, generalized hyperkeratosis of the palms and soles, and nail pachyonychia in Poikiloderma Clericuzio type (11). Compared to reticulate hyperpigmentation, which may fade, hypohidrosis and palmoplantar keratoderma are enduring characteristics of NFJ syndrome. DKC can also have similarities with NFJS but encompasses additional features such as alopecia, mucosal leukoplakia, poikiloderma, and blood dyscrasias (14). Table 4 shows the Pigmentation patterns and clinical manifestations of Inherited Reticulate Pigmentary Disorders.

**TABLE 4:** Pigmentation patterns and clinical manifestations of Inherited Reticulate Pigmentary Disorders.

Inherited reticulate pigmentary disorder	Pigmentation pattern and clinical manifestations
Naegeli-Franceschetti-Jadassohn syndrome (1–5)	Reticulate hyperpigmentation on the neck, chest, abdomen, and axillae, accompanied by hypoplasia of dermatoglyphics, dental anomalies, diffuse palmoplantar thickening, hypohidrosis, and nail dystrophy.
Dyschromatosis symmetrica hereditarian (6–8)	Mottled hypopigmented and hyperpigmented macules on the dorsal aspects of the extremities, along with congenital heart defects, hemangiomas, and neurological symptoms.
Dyschromatosis universalis hereditaria (27, 9, 10)	Irregularly sized and shaped mottled hyperpigmented and hypopigmented macules distributed randomly across the body, accompanied by sparse or thin hair, hypopigmented hair, and brittle hair and nails.
Reticulate acropigmentation of Kitamura (14, 16)	Angular, reticulate, freckle-like hyperpigmented macules on the dorsal aspect of the extremities, accompanied by epidermal atrophy.
Dowling-Degos disease (11, 13, 15)	Reticulate, dot-like hyperpigmentation in flexural areas and comedo-like follicular papules.
Dyskeratosis congenita (9, 12, 17, 23)	Congenital reticular hyperpigmentation, predominantly on the neck and chest, accompanied by leukoplakia and nail atrophy in both fingernails and toenails. Additionally, it is also associated with hematologic abnormalities, including aplastic anemia, myelodysplastic syndrome, leukemia, and bone marrow failure.
Dermatopathia pigmentosa reticularis (18–20)	Reticulate hyperpigmentation predominantly on the trunk, aplasia of dermatoglyphics, noncicatricial alopecia, hypohidrosis, and nail dystrophy.
X-linked reticulate pigmentary disorder (21, 22, 24, 28)	Males exhibit reticulate hyperpigmentation and hypopigmentation, an upswept frontal hairline, flared eyebrows, hypohidrosis, gastrointestinal inflammation, recurrent respiratory infections, and failure to thrive. Females typically show patchy pigmentation along the lines of Blaschko, without associated systemic manifestations.

Currently, no established treatment protocols are available for the NFJ syndrome. Management primarily focuses on addressing associated conditions. Most case reports and series included in this systematic review did not provide specific treatment details. Oral health maintenance is essential to prevent premature dental caries. Notably, doxycycline has demonstrated potential *in vitro* by interfering with TNF-a–mediated signaling and apoptosis, potentially holding promise for future therapeutic applications (1). Individuals afflicted with NFJ syndrome are encouraged to avoid strenuous physical activities, given that hypohidrosis represents the most debilitating aspect and can culminate in post-exertional collapse (14).

It is imperative to explore other treatment options. For other reticulate pigmentary disorders such as reticulate acropigmentation of Kitamura and DDD, it was observed that 20% azelaic acid ointment showed improvement in one patient following treatment after several weeks. For DSH, surgical therapy with transplantation of thin split-thickness skin autografts has been tried, whereas the keratoderma associated to DPR has been successfully treated with etretinate (26).

The current lack of pharmaceutical interventions and clinical trials in this domain underscores the existence of a significant research gap, underscoring the potential for future investigative studies. Although NFJS is a rare autosomal dominant ailment with an estimated incidence of one case in two to four million individuals, and currently shows no racial predilection (3, 14), it is crucial to recognize the significant impact on the quality of life of patients affected by this condition, primarily due to the noticeable dermatological manifestations and the associated challenges. In this context, serious consideration should be given to genetic testing and genetic counseling regarding NFJ Syndrome and a precise clinical diagnosis must be reached between the various types of reticular pigmentary disorders, especially for those families with an intense desire for future pregnancies.

## 5 Conclusion

NFJ Syndrome is a rare diagnosis that affects the skin, nails, teeth, and sweat glands. Our paper aims to provide insights into the clinical features, genetic mutations, and management of this condition. The information presented in this review could be useful for physicians in clinical practice to understand the clinical presentation and features of NFJ Syndrome.

## 6 Limitations and strengths

Our study presents the first systematic review summarizing the available clinical literature on NFJ Syndrome through case reports and case series. We have provided a comprehensive overview of published data, with a robust quality appraisal of the included studies. However, this systematic review has its limitations. Due to the limited literature on NFJ Syndrome, only case reports and case series were included, which may have introduced potential bias. Additionally, the absence of follow-up data in most cases hindered the evaluation of primary and secondary outcomes, limiting our ability to assess long-term clinical progression and management effectiveness.

## Data Availability

The original contributions presented in this study are included in this article/supplementary material, further inquiries can be directed to the corresponding author.
